# Urological knowledge and tools applied to diagnosis and surgery in deep infiltrating endometriosis – a narrative review

**DOI:** 10.1590/S1677-5538.IBJU.2023.9907

**Published:** 2023-05-30

**Authors:** André L. Lima Diniz, José Anacleto D. Resende, Cláudio M. de Andrade, Alice C. Brandão, Mauro P. Gasparoni, Luciano A. Favorito

**Affiliations:** 1 Universidade do Estado do Rio de Janeiro Unidade de Pesquisa Urogenital Rio de Janeiro RJ Brasil Unidade de Pesquisa Urogenital - Universidade do Estado do Rio de Janeiro - Uerj, Rio de Janeiro, RJ, Brasil

**Keywords:** Endometriosis, Urinary Bladder, Robotic Surgical Procedures

## Abstract

**Objectives:**

This review discusses deep infiltrating endometriosis (DIE) diagnosis and surgery using current urological knowledge and technologies.

**Materials and Methods:**

Narrative review of deep infiltrating endometriosis that result in urological issues. We examined manuscripts from Pubmed, Embase, and Scielo’s database using the following MeSH terms: (‘endometriosis’) AND (‘urology’ OR ‘urological’ OR ‘urologist’) AND (‘bladder’ OR’vesical’) AND (‘ureteral’ OR ‘ureter’). Selection followed PRISMA guidelines. Sample images from our records were brought to endorse the findings.

**Results:**

Thirty four related articles were chosen from 105. DIE may affect the urinary system in 52.6% of patients. Lower urinary tract symptoms may require urodynamic examination. Ultrasonography offers strong statistical yields for detecting urinary tract lesions or distortions, but magnetic resonance will confirm the diagnosis. Cystoscopy can detect active lesions, although any macroscopic visual appeal is pathognomonic. Endourology is utilized intraoperatively for bladder and ureteral assessment, however transurethral endoscopic excision of bladder lesions had higher recurrence rates. Laparoscopy is the route of choice for treatment;  partial cystectomy, and bladder shaving were the most prevalent surgical treatments for bladder endometriosis. Regarding the ureteral treatment, the simple ureterolysis and complex reconstructive techniques were described in most papers. Using anatomical landmarks or neuronavigation, pelvic surgical systematization allows intraoperative neural structure identification.

**Conclusions:**

DIE in the urinary system is common, however the number of publications with high level of evidence is limited. The initial tools for diagnosis are ultrasonography and cystoscopy, but magnetic resonance is the most reliable tool. When the patient has voiding symptoms, the urodynamic examination is crucial. Laparoscopy improves lesion detection and anatomical understanding. This approach must be carried out by professionals with high expertise, since the surgery goes beyond the resection of lesions and includes the preservation of nerve structures and urinary tract reconstruction techniques.

## INTRODUCTION

The presence of endometrial glands and stroma outside of their normal anatomic location was first described by Rokitansky in 1860 (1), and the first publications on this topic are about to complete their first centenary. In 1921, Sampson described the hemorrhagic chocolate cysts (2) and a few years later, in 1925, coined the name endometriosis (3). The disease is now understood as the presence of endometrial glands and stroma outside the uterine cavity.

According to epidemiological researches, the general prevalence is usually estimated to be 10% among women who are fertile (4) and despite the fact that it can afflict youngsters (5, 6), the highest occurrence is often documented between the ages of 25 and 35 years of age (7, 8). The average annual incidence rate is about 7.2 per 10,000 (9) and although this data may have variations, patterns show a reduction following the end of women’s reproductive age, with a drop just after the age of 45 (8).

Usually related to pain, menstrual disorders, and infertility, it is a disease that can show up in different ways. It has the potential to stand as superficial implants on the peritoneal surface, been ovarian cysts called endometriomas or advance as lesions that infiltrate the muscularis propria of surrounding organs or even penetrate the peritoneal surface by more than 5 mm. Of these three possible phenotypes, this last one is the most aggressive; known as deeply infiltrating endometriosis (DIE) it is typically, but not solely, found in the pelvic compartment (10).

The anatomical obviousness would make the naivest observer relate this entity only to gynecology. Endometriosis is highly unpredictable and can manifest in the most random locations including head (11, 12), thorax (13–16), extra-pelvic abdominal organs (17–22) and the abdominal wall itself (23, 24). This wide range brings the newbie back to the real world, but it must be said that atypical DIE mostly affects the gastrointestinal and urinary tract (25). In this way, care will be better guided by a team composed by a gynecologist, coloproctologist/digestive system surgeon, and urologist. The purpose of this article is to describe urological knowledge and tools applied to diagnosis and surgery in deep infiltrating endometriosis.

## MATERIALS AND METHODS

In this study we carried out a review about the urological knowledge and tools applied to diagnosis and surgery in deep infiltrating endometriosis. We analyzed papers published in the past 20 years in the databases of Pubmed database (US National Library of Medicine, Bethesda, Maryland), Embase and Scielo, found by using the following combination of MeSH terms: (‘endometriosis’) AND (‘urology’ OR ‘urological’ OR ‘urologist’) AND (‘bladder’ OR ‘vesical’) AND (‘ureteral’ OR ‘ureter’). The linked articles algorithm on Pubmed was used to identify English language and peer-reviewed journal articles published.

Original articles related to diagnosis, papers describing anatomy issues of the pelvis related to urological organs, descriptions of surgical techniques and outcomes were included if based on urinary tract matter. Clinical studies on complications functional urogenital disorders in endometriosis have also been analyzed. Studies with the following designs were included: randomized clinical trials, pre-post intervention studies and observational (cohort and case-control) studies assessing women of reproductive age with DIE involving urological structures. Clinical studies of non-urological complications were excluded. Related documents with communications in congress, reviews, opinions, case reports and case series with less than 10 patients have been also excluded. No attempt was made to identify the “gray literature”. After screening, articles were first considered eligible by their title and abstract. Records were then evaluated in full articles before being included to the qualitative synthesis group. Bibliographies of essential articles were also studied to find papers of additional interest. PRISMA guidelines were followed during the selection process (26).

Authors provide sample imaging from his own database in support of the data found.

## RESULTS

**Figure 1:**
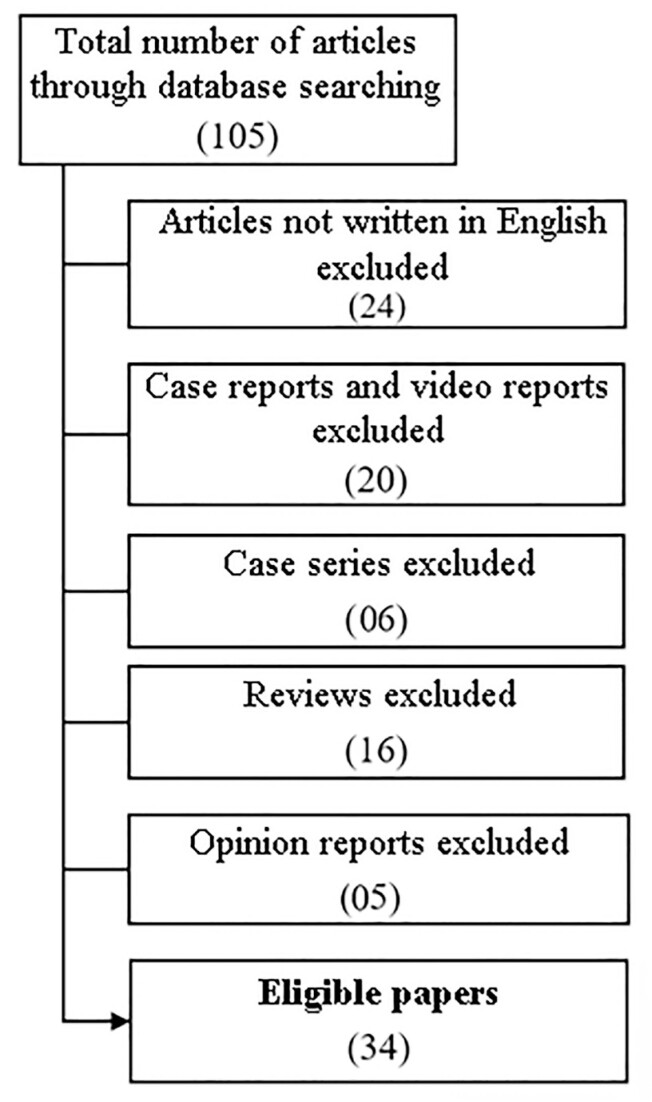
Flow diagram of the literature search.

A total of 105 articles were identified as potentially relevant. In the first hand, 24 papers were excluded for being written in other languages than English, of these four were case reports, one reported case with less than ten patients enrolled and one was a literature review. The following criteria disqualified English-language papers that did not meet the inclusion requirements. In this sense, 20 case reports—of which half had videos attached—were disregarded. Six studies reported series of cases involving less than ten participants, thus these were also eliminated. Sixteen studies were pure literature reviews; thus, they were eliminated. Finally, five publications describing expert opinions were disqualified according to the requirements. The flow diagram is depicted in Figure-1.

Considering the 34 publications selected, we organized the content by research design and issues pertinent to the prognosis of deep infiltrating endometriosis. Our search yielded three retrospective studies and two prospective cohort studies that assessed radiological diagnostic techniques. One of them also analyzed the cystoscopy as a diagnostic tool and other three retrospective studies have detailed this endoscopic route employment. Three of those diagnostic studies also reported on clinical patient outcomes. Seven cohort studies, twelve retrospective analyses, and nine case series reported surgical procedures and/or clinical outcomes. There were no randomized clinical trials found, only observational research.

### Descriptions of the radiological diagnostic techniques

Three of the five investigations on the radiological diagnosis of DIE included ultrasonography (US), while the other two involved magnetic resonance imaging (MRI).

**Figure 2:**
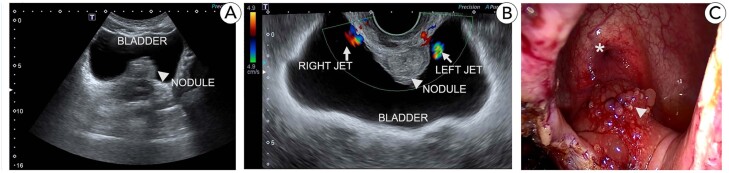
Nodule (arrowhead) identified at transabdominal US (A). Transvaginal US (B) was able to demonstrate bilateral urethral patency using doppler (arrows) despite the proximity to the nodule (arrowhead). Laparoscopic view (C) of the nodule (arrowhead) and bladder trigone (asterisk) just before partial cystectomy.

The accuracy of transvaginal ultrasonography (TVUS) in predicting detrusor infiltration and ureteral meatus involvement in individuals with bladder endometriosis (BE) was examined by Ros and colleagues in 2021 TVUS detected bladder wall BE nodules and accurately predicted cystoscopic results in their sample; 21 of 22 women had uninvolved ureteral orifices at least 10 mm from the BE nodule (TVUS sensitivity, 95%). Authors concluded that cystoscopy may be unnecessary for partly muscular nodules seen at TVUS, as the example in Figure-2. Having said that, the researchers are aware of the limited statistical power of their findings related to the reduced number of participants enrolled (27). Years before, in a retrospective study, Hudelist et al. could evaluate the TVUS for preoperative detection of BE in a larger sample of which fifty of 207 patients with DIE had urinary endometriosis, including 30 patients with BE and 23 women with either isolated or multiple hydronephroses. TVUS was able to find bladder endometriosis with a sensitivity of 93%, a specificity of 99%, a positive and negative predictive value of 99% and 97% respectively and a test accuracy of 98.6% (28).

These authors’ findings are in line with the conclusions of a cohort study conducted in 2015 by Pateman and coworkers who highlighted that US is an accurate test to diagnose urinary tract involvement in women with suspected pelvic endometriosis. Over the course of 14 months these researchers followed 848 women with chronic pelvic pain in whom suspicion of DIE was raised. One major concern of these researchers was to evaluate the effects of DIE on the upper urinary tract. So, in addition to USTV patients also underwent sonography of their kidneys. Given the service’s tertiary nature, an unexpected epidemiology was found. US was able to show endometriosis in 335 women (39.5% 95% CI 36.2-42.8). Bladder seeds were observed in 6 women. Three patients had both, ureteric and bladder endometriosis. All bladder nodules were attached to the anterior uterus, above the trigone, medial to the ureters and distant from ostia; three were in the midline, two were to the left, and one was to the right. Ureteric endometriotic lesions were seen in 14 cases (8 on left, 6 on right). Of those fourteen women with ureteric involvement, two were referred to that specialized unity due to previous known hydronephrosis. The further statistical analysis was then made with a sample of 12 patients. Ten of these 12 subjects had ipsilateral upper urinary tract dilatation on transabdominal US and the other two women had distal ureteric distension without hydronephrosis. Pre-operative sonography for ureteral DIE showed a sensitivity of 92.3% (95% CI 63.9-99.8), specificity 100% (95% CI 97.6–100), PPV 100% (95% CI 73.5-100), and NPV 99.3% (95% CI 96.3–99.9%) (29).

Despite the previously demonstrated high statistical yields of ultrasonography, the recognition of a urinary tract lesion or distortion is simply a first step that alerts the surgeon to the disease’s complexity. Data from Patemans’s paper has shown that almost 58% of woman with endometriosis had lesions at two or more different locations using sonography (29). In this scenario, an MRI will then be critical to obtain a greater understanding and plan for the future of the procedure, since a US alone may not be quite enough.

**Figure 3:**
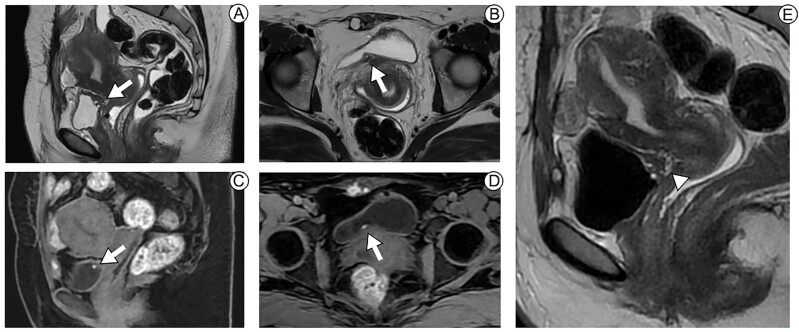
MRI shows DIE at the posterior aspect of the bladder (arrows), in the trigone closer to the right ureteral ostium. MRI sequences are T2-weighted at images A, E (sagittal plane) and B (axial plane). MRI sequences are T1-weighted at images C (sagittal plane) and D (axial plane). Late MRI sequence was performed (E) to allow bladder distension (note the bladder hypointense signal intensity related to Gadolinium contrast media), improving the lesion - ureteral distance measurement. There is muscle and mucosal infiltration.

Our search identified a retrospective study that evaluated the performance of MRI in locating endometriosis implants within the bladder wall with assessment of ureteral orifice extension using surgical findings as standard of reference. Researchers from academic medical centers had 39 exams by two senior members of the team. The mean size of the endometrioid foci was 30 ± 7 [SD]mm (range 19-41 mm); 56% were found in the median of the bladder wall, 26% left sided and 18% right sided. Still on the location of the lesion, 87% endometriosis implants were present in the two anterior thirds of the dome (sensitivity 100%; specificity 83%, accuracy 97%), 79% extended or were present in the posterior third pouch (sensitivity 100%; specificity 88%; accuracy 97%) and 64% extended into the bladder base (sensitivity 90%; specificity 87%, accuracy 89%). No extensive nodular endometriosis orifice infiltration was reported. The mean distance measured from the implant to the ureteral orifices was 9 ± 9 [SD] mm (range: 0–28 mm) at right, and 12 ± 12 [SD] mm (range: 0–55 mm) for the left one. Nine of these 25 patients with bladder base involvement, had a zero-distance reported between endometriosis implants and ureteral orifices, all but one presenting with low-to-moderate bladder volume repletion; only two of those nine women had ipsilateral ureteral dilatation on MRI. In addition to demonstrating the high accuracy of the method, Rousset et al. point out that adequate bladder filling, as shows Figure-3, is needed to improve appropriate estimate of the distance between endometriosis implants and ureteral orifices to better predict requirement of ureteral resection-reimplantation (30).

The importance of standardizing MRI studies highlighted by the previous author was also mentioned in a cohort study identified in our search. According to Bielen and colleagues, the capacity to determine the involvement of an organ system is highly dependent on the techniques adopted. In a tertiary care academic center 74 women were enrolled and the accurateness of the preoperative examination of specific organ systems using TVUS, double-contrast barium enema (DCBE), intravenous urography (IVU), and MRI was assessed. Regarding the urinary tract, the bladder involvement was detected in 10 individuals during laparoscopy. This was determined accurately by TVS in six individuals; however, the invasion depth was wrongly calculated in several instances (muscular vs submucosal or vice versa). However, the involvement of the bladder wall was appropriately identified on MRI in just two cases and was underestimated in one patient. Ureteral invasion was evaluated with IVU and MRI. The assessment of DE lesions in ureters was correct in 89,2% of patients based on IVU and 91,9% based on MRI. Pelvic ureteral encasement (i.e. narrowing with smooth wall lining) was not present in any of the patients at laparoscopy, though it was suggested in two patients based on IVU and/or MRI. Pelvic ureteral displacement was present in 28 patients and was detected by IVU in 57.1% of patients while MRI detected the displacement in an additional nine patients, resulting in a detection score of 89.3% (31).

### Descriptions of the endoscopy diagnostic techniques

**Figure 4:**
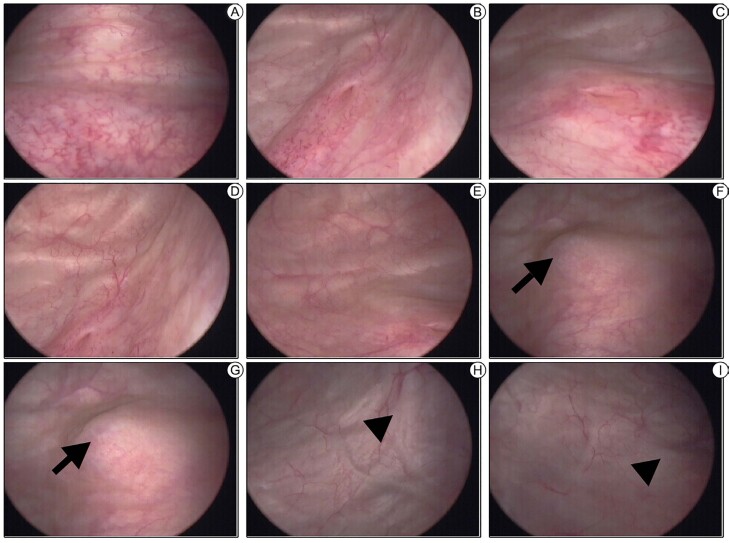
Cystoscopy shows bladder trigone (A), left (B) and right (C) ureteral ostia, left (D) and right (E) lateral walls free of lesions. A nodule (arrow) measuring approximately 1.5cm was found on the posterior wall of the bladder (F and G) at approximately 9mm from the right ureteral orifice and 11mm from the left ureteral orifice; bladder projection (arrowhead) suggests thickening of the left (H) and right (I) round ligaments.

At the previous session, data from Ros et al. were brought; in their institution, flexible cystoscopy was performed as routine for all women with US suspicion of BE affecting the bladder muscular layer. In their study, it was concluded that cystoscopy may be unnecessary for partly muscular nodules seen at TVUS (27), but authors cites a publication in which recognizes that for infiltrative BE nodules, cystoscopy is able to detect the distance from the BE nodule to the ureteral meatus and the trigone (32), as demonstrated in Figure-4.

**Figure 5:**
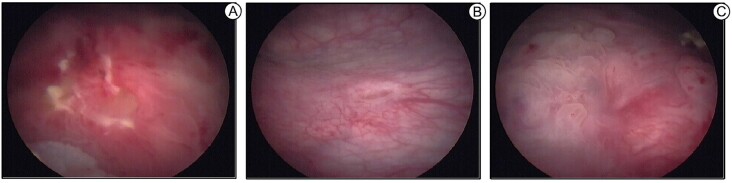
Bladder infiltrative lesion at the bladder trigone (A), 1.5 cm far from left ureter orifice (B); right ureter orifice (C) with infiltrative lesion. Upon vaginal examination, it was possible to perceive the presence of a palpable intravaginal nodule, adhering to the right intramural ureter.

In a retrospective study which enrolled 22 patients, Schneider et al observed internal bladder endometriosis in 15 women (68.1%), cystoscopy revealed that its location was at the dome in nine patients (40.9%), at the base in five patients (22.7%), and multifocal in one patient (4.5%). In the same population, IVU only suggested the disease in four cases (18.1%). They quote a traditional statement from the literature on the subject that reminds the reader to repeat cystoscopy at different times of the menstrual cycle, since endometriosis is best diagnosed before and during menstruation. Their study shows that cystoscopy is important for the potential diagnosis of DIE, the authors make it clear that the macroscopic aspect is not pathognomonic, even in cases as shown in Figure-5 (33).

Antonelli and cols. analyzed their database and found 1242 patients with surgically proved diagnosis of endometriosis within ten years; it was found that 31 patients (2.5%) had urinary tract involvement. The bladder was affected in 12 patients, the ureter in 15, and either the bladder or the ureter in 4. Cystoscopy was performed in all cases with bladder endometriosis and found the typical bluish irregular submucosal lesion on the dome (8 patients) or base (8 patients) of the bladder. Of the 16 cases in which cystoscopy corroborated the diagnosis of BE, transurethral endoscopic resection was performed in two patients with mean lesion size of 1.9cm, both women had bladder recurrence. Six individuals received open partial cystectomy and mean lesion size was 3.5cm; four cases were treated with laparoscopic partial cystectomy and mean lesion size was 3.6cm. Three patients underwent ureterocystoneostomy and partial cystectomy, bladder mean lesion size was 1.5cm. One woman was treated with laparoscopic bilateral ureterolysis and partial cystectomy, the lesion measured 3cm. None of these 14 subjects faced bladder recurrence. In face of their findings Antonelli et al. states that cystoscopy is advisable in women affected by endometriosis complaining LUTSs or haematuria; and reinforces that an ultrasonographic study of the upper urinary tract should be performed in all patients with pelvic endometriosis, even in the absence of urological symptoms (34).

**Figure 6:**
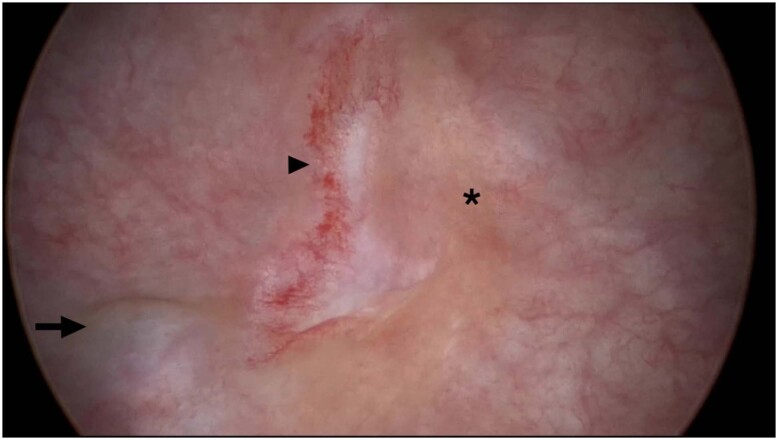
Cystoscopy identifies bladder endometriosis (arrow) and thermal injuries related to hysterectomy (arrowhead: damage to the inner layer of the muscularis; asterisk: mucosal damage).

By furthermore to the preoperative diagnostic, cystoscopy was found as an intraoperative method for bladder and ureteral evaluation. Some authors advocate the employment of cystoscopy during hysterectomy for benign causes as a tool for the prompt diagnosis of potential iatrogenic damage as perceived in Figure-6 (35, 36).

**Figure 7:**
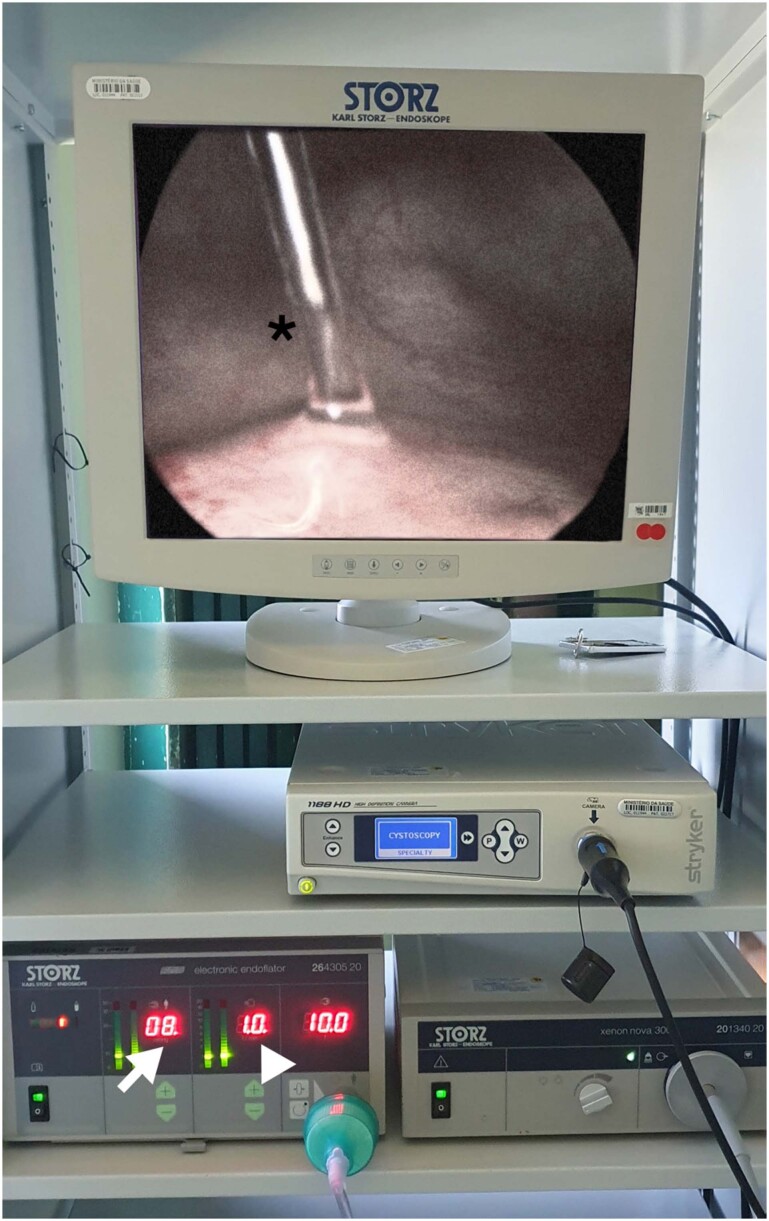
CO_2_ cystoscopy using a laparoscopic insufflator at pressure of 8mmHg (arrow), flow rate at 1L/min (arrowhead); urine jet identified (asterisk).

Oliveira et al. described the perioperative cystoscopy in 47 patients just after pelvic surgeries, 26 women (55%) had laparoscopic hysterectomy and 21 (45%) underwent bladder endometriosis nodule resection; among these last, 13 patients had the shaving dissection (without the need to open the mucosa, but with detrusor suturing) and 8 patients underwent partial cystectomy. This cystoscopy was performed under CO_2_ filling with constant pressure of 8 mmHg and flow of 1L/min; as demonstrated in Figure-7, kindly provided by Dr. Oliveira.

Regular cystoscopy filling the bladder with saline solution may impose some difficulty to jet identification due to the urinary density and the use of dyes may cause adverse effects. This cystoscopy using CO2 allows the evaluation of the anatomy of ureteral orifices, the presence of ureteral jets and the integrity of bladder walls, with the same acumen as the regular fashion; the mean time between the onset of the cystoscopy and the observation of bilateral jets were 145 seconds, suggesting their patency. In face of their findings, Oliveira and colleagues stated that the cystoscopy technique using CO2, was fast, easy, safe and efficient (37).

### Descriptions of the surgical procedures and clinical outcomes

Six of the publications selected for the aforesaid purpose described diverse etiologies and/or therapy of urinary tract lesions. All these resources reported endometriosis, but without pushing our topic. Due to its worth, certain findings were brought to this study.

**Figure 8:**
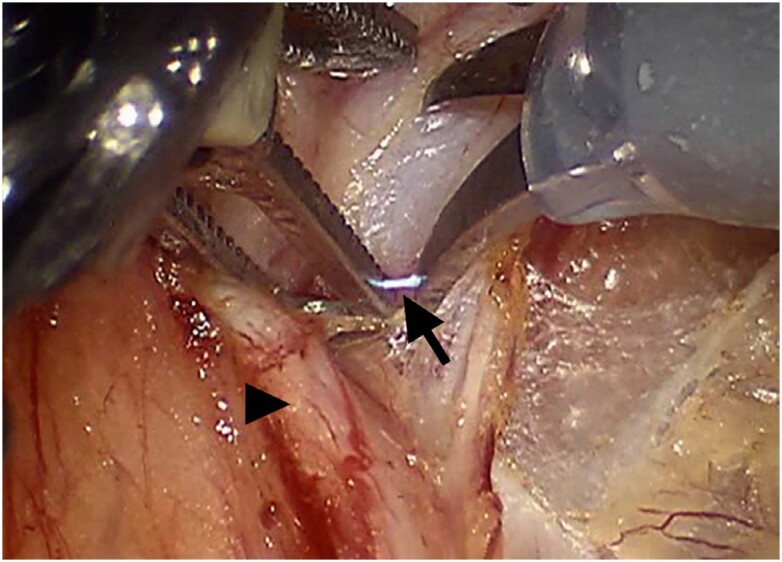
Electrical conduction between monopolar scissor and grasper (arrow) during lymphadenectomy step. Grasper is pulling the ureter (arrowhead) away from the dissection area.

In a cohort study, Dallas et al. analyzed a sample of 296,130 women who underwent a hysterectomy procedure for benign conditions, which 18.8% were related to endometriosis. When such diagnosis was present, genitourinary injuries happened in about 2.3% of the women demonstrating an increased likelihood (OR 1.46; 95% CI 1.36-1.56) of a genitourinary injury occur, with a delayed diagnosis happening in about 20.6% of the cases (36). These results were in accordance with a retrospective performed by Wallis and colleagues who similarly found that a primary post-operative diagnosis of endometriosis was associated with a significantly increased risk (OR 1.92, 95% CI 1.68-2.19) of urinary tract injury (35). Johnston et al. reported that 28% of their 1265 laparoscopic procedures were for endometriosis treatment, but happening only two urinary tract damages (38). With a smaller sample, Siow et al. studied 495 cases of laparoscopic hysterectomy, 25,1% due to endometriosis; urological lesions were found in 1.6% of patients; authors suggests that the most likely etiology was thermal damage from electrocautery used to secure hemostasis of the uterine artery pedicle and consider the endometriosis as one of the risk factors related (39). Inadvertent electrical conduction between instruments and tissues can also occur (Figure-8) and, when identified, should lead to revision of the affected tissue, as well as strict follow-up of the patient in the postoperative period, given the risk of late leakage.

**Figure 9:**
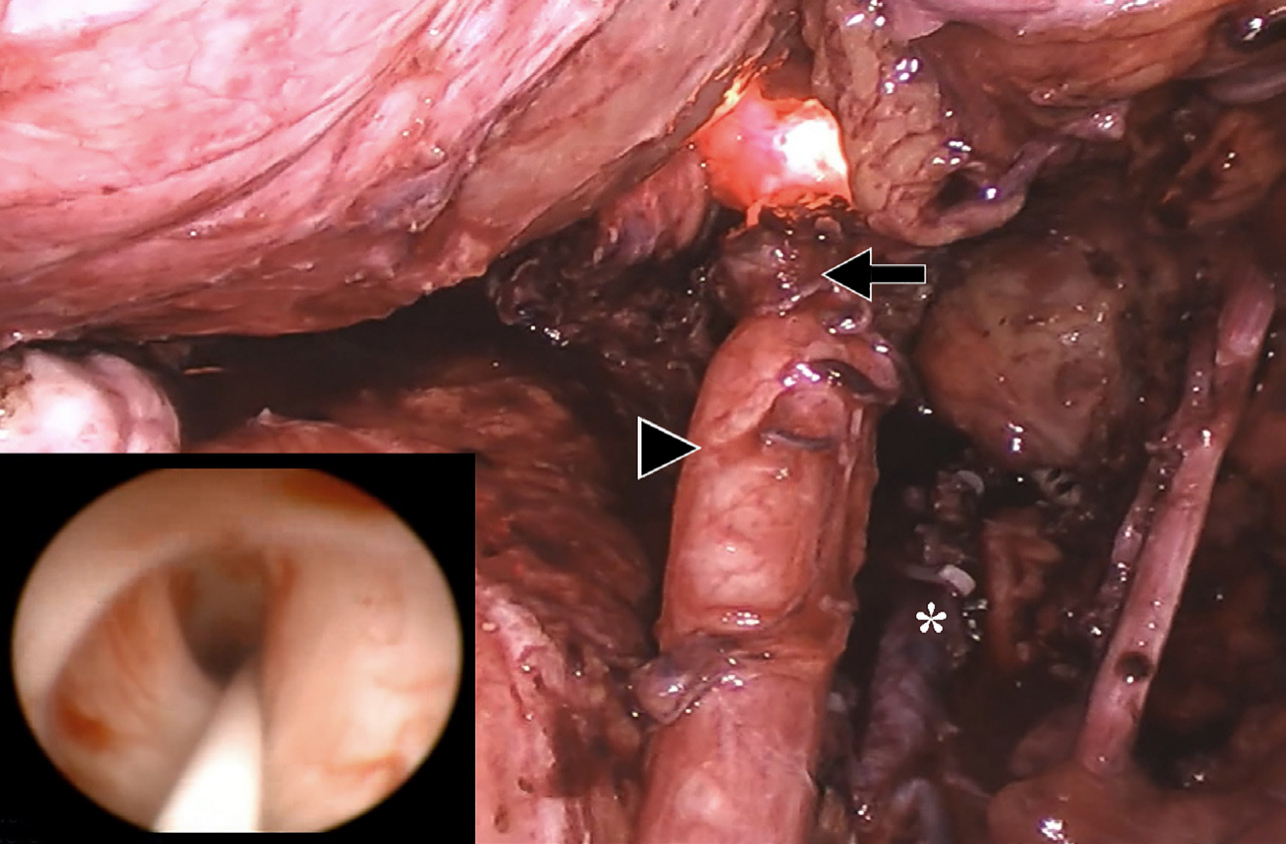
Double endoscopic view of an extrinsic endometriotic lesion at the distal right ureter. A nitinol guidewire was used to allow safe progression of the semi-rigid ureteroscope as shown. The arrow shows laparoscopic aspect of the endometriosis, and the arrowhead shows the ureteral distension above the stricture point. Uterine artery, marked with asterisk, was ligated with Hem’O’Lock clips.

Two case series documented ureteral stricture surgery for distinct causes. Five of our 44 patients experienced endometriosis-related ureteral stricture, similar to that shown in Figure-9. In four patients, the distal constriction was treated by ureteroneocystostomy (UNC) with a Boari flap and two with UNC with the psoas hitch approach. UNC with a Boari flap was used to treat a 80mm lesion in the middle-inferior ureter transition. Both strategies work long-term without concerns (40, 41). This favorable outcome was also observed in a cohort study by Carmignani et al., who evaluated thirteen patients with deep endometriosis and ureteral involvement and concluded that the combination of bladder psoas hitch, ureteral resection, and ureteroneocystostomy had no negative effect on urodynamic parameters (42).

In order to increase understanding of the uro-neurology interaction, Chiantera et al. presented the use of neuronavigation methods for the treatment of deep endometriosis in a retrospective investigation. Involvement of deep lateral tissues was detected in 40% of women with central pelvic lesions and in 72.7% of patients with involvement of the hypogastric plexus. The authors concluded that complete laparoscopic excision of endometriosis tends to reduce recurrence rates without increasing surgical morbidity (43).

Twenty-two studies provided relevant evidence that primarily highlighted the proposed issue; however, the non-uniform description of clinical information, treatments, and results hinders the detailed analysis of the pooled data. Two of those papers referred exclusively to ureteral endometriosis.

For the treatment of bladder endometriosis, the techniques described were transurethral endoscopic resection (TURB), partial cystectomy with mucosal preservation (shaving) and partial cystectomy. Partial cystectomy was the most common surgical treatment for endometriosis of the bladder, as reported in twenty studies. A total of 1,355 patients were enrolled, and in addition to several further ureteral procedures, 598 partial cystectomies (44.13%) were done.

The use of TURB was documented in six studies with a total of 149 bladder endometriosis patients, and thus the endoscopic approach was adopted in 35% (52 patients). Recurrence was identified in 24 women, which corresponds to an estimated recurrence rate of 46.15%. Data are described in Table-1. The National Survey conducted by Hirata and colleagues stands out among the reported research, which cumulative recurrence rates were considerably greater with TURB than with partial cystectomy under statistical significance (p < 0.05).

**Table 1:** Patients with bladder endometriosis treated by transurethral endoscopic resection.

Authors	Bladder endometriosis	TURB	Recurrences
Hirata et al.(44)	89	34	19
Freire et al. (45)	15	3	0
Schneider et al. (46)	15	5	0
Antonelli et al. (34)	16	2	2
Kumar et al. (47)	9	5	2
Perez et al. (48)	5	3	1
**Total**	**149**	**52**	**24**

**Figure 10:**
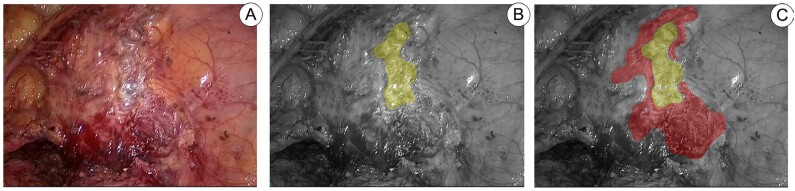
Image A shows bladder after laparoscopic resection of endometriosis, highlighted in yellow at Image B, the area delimits the resection performed up to the mucosa and marked in red at image C, we demonstrate the bladder resection in which it was possible to preserve the muscle layer.

In several investigations, removal of bladder nodules maintaining the mucosa was also documented. This method has also been referred to as bladder shaving and bladder skinning, as demonstrated in the following series at Figure-10.

**Figure 11:**
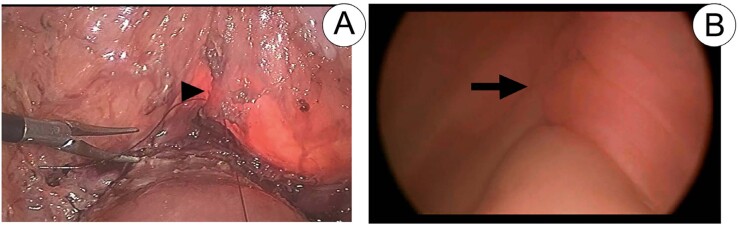
Cystorraphy line (arrowhead) demonstrated by laparoscopy at Image A and by cystoscopy (arrow) at Image B. Bladder filling during cystoscopy also makes it possible to assess the watertightness of the suture performed.

As described by Darwish et al., this technique begins with a detrusor incision around the bladder lesion, followed by dissection to the nodule’s macroscopic limits in the depth, and as resection appears complete prior to opening the bladder, muscular suture in one layer to reinforce the bladder wall is then performed (44). With data presented in Table-2, by examining these publications, there have been 109 people with vesical endometriosis; and 44 of them had bladder shaving, which corresponds to about 40.37 percent of cases; just one patient out of 44 experienced recurrences, which amounts to 2.27%. In these areas of weakening of the full thickness of the bladder, reinforcing suture is suggested, as shown in Figure-11.

**Table 2:** Patients with bladder endometriosis treated by bladder shaving.

Authors	Bladder endometriosis	Shaving	Recurrences
Maida et al. (50)	21	4	1
Alves et al. (51)	5	3	0
Darwish et al. (49)	50	15	0
Gabriel et al. (52)	33	22	0
**Total**	**109**	**44**	**1**

Ureteral involvement by endometriosis foci may need various types of surgery; among the publications assessed, less invasive resection procedures such as ureteroscopic approaches to simple ureterolysis (N = 282), progressing to segmental ureterectomy with end-to-end anastomosis (N = 70), UNC as Lich-Gregoir or Politano-Leadbetter (N= 271), UNC with Boari flap (N = 4), and UNC with psoas hitch (N = 17). Some combined procedures have been described, one ureteric stump was resected and a bladder cuff, two PC with ureterolysis, four PC plus ureterectomy with end-to-end anastomosis and four PC added to UNC as Lich-Gregoir. Six nephrectomies were performed due to loss of renal function related to ureteral obstruction by endometriosis.

## DISCUSSION

Endometriosis reveals itself in a variety of clinical manifestations, and the urologist is rarely the first health care provider to interact with the patient. Menstrual disorders, chronic pelvic pain, and infertility are typically incorporated into the differential diagnosis for endometriosis. The combination of symptoms and signs enables us to properly demand imaging studies. As shown in the literature, sonographic evidence can eventually be used to make a presumptive diagnosis of the condition and evaluate the upper urinary tract; however, MRI scans are most often robust for the nonsurgical diagnosis of endometriosis.

However, if the patient discloses symptoms of the lower urinary tract during the interview and radiological findings corroborate the diagnosis of endometriosis, a urological consultation is required. Urodynamic and endoscopic examination may be requested in this case.

Researchers found that 96.7% of patients in a prospective study with 30 patients had one or more abnormalities at urodynamic examination; and those women with anterior compartment endometriosis had increased bladder sensation (90.0% versus 45.0%, p=0.024) and painful bladder filling (70.0% versus 30.0, p=0.04) compared to patients with posterior endometriosis only (45). In line with the results of this study, another team of investigators accessed data at a considerably larger population of 138 women with radiological diagnosis of endometriosis and found that the harm of the bladder was a statistically significant independent predictor of low bladder compliance, whereas endometriosis in the parametrium was a statistically significant independent predictor of both abnormal residual urine and bladder outlet obstruction (46).

Even though it is invasive, cystoscopy is inexpensive, useful for estimating the distance between ureteral orifices and nodule boundaries and may allow biopsy if necessary. However, due to the intraperitoneal origin of the nodule, traditional outpatient cystoscopy is more usually normal; in only half of the instances, a classic adenomatous and nodular red or bluish mass expansion is visible, and ulcerations are more rarer (27, 47). In this sense, cystoscopy under sedation concomitant with physical examination of the pelvis, including bimanual palpation of the bladder, has been proposed to improve the accuracy of the method. By using this dynamic cystoscopy in a study of 157 participants, researchers perceived that the test’s had a high specificity (97.78%) and low sensitivity (58.21%) with substantial positive predictive value (95.12%) and negative predictive value (75.86%); this study has also shown that abnormalities during dynamic cystoscopy were associated with a higher ratio of bladder surgery for the treatment of deep endometriosis, and typical findings involving the mucosa tends to be associated with a higher ratio of partial cystectomy (48).

Depending on where the endometriotic lesions are located, deep pelvic endometriosis is anatomically classified as anterior, middle, and posterior compartments. The anterior compartment is the region just posterior to the pubic symphysis, where endometrial implants may happen within the vesicouterine pouch, vesicovaginal septum, bladder, and ureter. The middle compartment consists of the uterus, fallopian tubes, ovaries, mesovarium and broad ligaments. The posterior compartment is located between the posterior vaginal wall and the anterior rectal wall, it consists of the rectovaginal pouch, rectocervical space, the rectovaginal sep­tum, uterosacral ligaments (USL), as well as the rectosigmoid (49). Disease distribution may also be classified as: Central Pelvic Endometriosis (CPE) when DIE involved one of these anatomic sites: cervix, vagina, uterosacral ligaments, rectum, bladder and pelvic peritoneum; superficial Lateral Pelvic Endometriosis (sLPE) when parametria, ureters or hypogastric plexus were involved; deep Lateral Pelvic Endometriosis (dLPE) in presence of sacral plexus and/or sciatic nerve infiltration (50).

The frequency of urinary tract involvement due to DIE may reach numbers as high as 52.6% as found in our search (51). The bladder is the most frequently affected organ in the urinary system and the endometriotic foci are usually found at the dome but it may extend to its base and even get close to the ureteric ostium (30, 31). During laparoscopy surgeries, a cystoscopy may assist partial laparoscopic cystectomy with a light-to-light methodology which enables appropriate intra- or extravesical detection of lesion boundaries (52). It may permit a sparring surgery in an effort to proceed the partial cystectomy or even a bladder shaving as described by Darwish (44). Still debating about minimally invasive approaches, the ureteroscopy may also be useful if ureteric lesions are presumed or suspected. Freire et al. reported 3 endoscopic management of ureteral lesions (53); in the same sense, Kumar described the diagnosis of DIE of the ureter in one patient of his sample (54).

In the pelvis, endometriotic lesions are more frequently observed in the posterior compartment and on the left side (55). The uterosacral ligament and rectum are the most affected structures (43). The sigmoid colon promotes retrograde menstrual fluid stasis on the left, sheltering and inhibiting endometriotic cell diffusion from the left hemipelvis; hence, the left ureter is more often implicated than right, as shown by Ceccaroni et al. in his cohort study which found 151 patients with unilateral ureteral stenosis, 39 patients in the right ureter and 112 patients in the left (56). These lesions usually occur above the ureterovesical junction, where the ureter crosses the uterine artery. Large paracervical or pararectal lesions larger than 2 cm may involve the ureter (57).

An extensive ureteral involvement by DIE can result in hydronephrosis and asymptomatic loss of renal function, as demonstrated by Antonelli et al, who performed two nephrectomies for end-stage renal atrophy (34) and by Freire et al, whose patient populations with ureteral involvement had hydronephrosis, of which 41.2% (7/17) had partial and 17.7% (3/17) had total loss of renal function (53).

The surgeon who intends to act on the pelvis must comprehend ureteral fragility. This retroperitoneal tubular structure is harmed more frequently during gynecological pelvic operations (36, 37, 58). Not even the preemptive stenting is able to avoid harm as described by Siow et al. which states that the presence of the ureteric stents may actually make the ureter less pliable and more rigid thereby increasing the risk of injury during dissection to mobilize it (58).

The initial anatomical landmark in the systematic surgical treatment of endometriosis is the cranial pelvic point close to the promontory, below the split of the cava and aorta into iliac vessels. Access to the left ureter begins with sigmoid release from parietal peritoneal adhesions. Identification of the left ureter in the retroperitoneum in a healthy area, typically crossing the common iliac artery, enables ureterolysis in a distal direction, clearing the lateral aspect of the organ from the uterosacral ligament and the peritoneum of the ovarian fossa, which are frequently affected by endometriosis foci. Returning to that initial landmark, usually crossing the external iliac artery, we can find the right ureter covered by peritoneum; and as with the contralateral one, the release of adhesions and endometriotic foci from the ovarian fossa and uterosacral ligament allows safe mobilization of the ureter. Ureterolysis of the medial face should be avoided since the vascularization of the distal portion may be compromised. Among the enrolled manuscripts, some provided a comprehensive explanation of surgical steps, with special mention to Abo et al., Darwish et al., and Maida et al.; the last adds supplemental video content to the papper (44, 59, 60).

Although they are extra-peritoneal structures, deep infiltrating endometriosis can harm the pelvic nerves. While this is not a signaling route unique to the urinary tract, it is imperative that the urologist be familiar with the treatment and dissection of these structures since those nerves are necessary for the proper functioning of the lower urinary tract.

The surgical systematization of the pelvis enables the intraoperative identification of neural structures based on knowledge of anatomical landmarks. On occasion, neuronavigation techniques may be advantageous, particularly when structures requiring neurolysis are at danger of being accidentally damaged during dissection (43). We can identify the motor response of a tissue that is thought to be a nerve by applying bipolar current to it, so that connections with S3 produce flexion of the hallux and contractions of the pelvic floor, rectum, and bladder, while connections with S2 cause flexion of the other toes. Damages to the superior hypogastric nerves may lead to urinary retention, urinary hesitancy, constipation, urinary and fecal incontinence, and sexual dysfunction; inferior hypogastric plexus harm is often related to urinary retention, constipation, and sexual dysfunction; the injury of pelvic splanchnic nerves may also cause urinary retention (61, 62). Mastering these concepts is essential for preventing bladder hypotonia or atony in the future, which is of utmost importance in urology.

## CONCLUSIONS

Even experienced surgeons find pelvic surgery challenging. This paper emphasizes the role of the urologist in deep infiltrating endometriosis care.

The risk of inadvertent injury to urinary tract structures can be minimized with proper training and anatomy understanding. The diagnostic methods of endometriosis are represented by USTV, MRI and cystoscopy. This endoscopic tool can also be useful during surgeries. Although bladder involvement is more common, ureteral injuries may be asymptomatic and cause long-term renal dysfunction. Surgical treatment of EID has been mainly performed by conventional and robotic-assisted laparoscopy. The use of systematic surgical steps allows the identification and preservation of nerves and surrounding tissues. Bladder sparing tactics may be relevant for the future voiding pattern of the patient, however the transurethral resection approach is related to high rates of recurrence. In order to effectively treat ureteral injuries, it is necessary to achieve a balance between gentle ureter manipulation and skillsets of urinary reconstructive procedures.
